# Simultaneous Assessment of Cardiomyocyte DNA Synthesis and Ploidy: A Method to Assist Quantification of Cardiomyocyte Regeneration and Turnover

**DOI:** 10.3791/53979

**Published:** 2016-05-23

**Authors:** Gavin D. Richardson

**Affiliations:** ^1^Institute of Genetic Medicine, International Centre for Life, Newcastle University

**Keywords:** Developmental Biology, Issue 111, Cardiomyocyte, regeneration, turnover, ploidy, flow cytometry, heart

## Abstract

Although it is accepted that the heart has a limited potential to regenerate cardiomyocytes following injury and that low levels of cardiomyocyte turnover occur during normal ageing, quantification of these events remains challenging. This is in part due to the rarity of the process and the fact that multiple cellular sources contribute to myocardial maintenance. Furthermore, DNA duplication within cardiomyocytes often leads to a polyploid cardiomyocyte and only rarely leads to new cardiomyocytes by cellular division. In order to accurately quantify cardiomyocyte turnover discrimination between these processes is essential. The protocol described here employs long term nucleoside labeling in order to label all nuclei which have arisen as a result of DNA replication and cardiomyocyte nuclei identified by utilizing nuclei isolation and subsequent PCM1 immunolabeling. Together this allows the accurate and sensitive identification of the nucleoside labeling of the cardiomyocyte nuclei population. Furthermore, 4′,6-diamidino-2-phenylindole labeling and analysis of nuclei ploidy, enables the discrimination of neo-cardiomyocyte nuclei from nuclei which have incorporated nucleoside during polyploidization. Although this method cannot control for cardiomyocyte binucleation, it allows a rapid and robust quantification of neo-cardiomyocyte nuclei while accounting for polyploidization. This method has a number of downstream applications including assessing the potential therapeutics to enhance cardiomyocyte regeneration or investigating the effects of cardiac disease on cardiomyocyte turnover and ploidy. This technique is also compatible with additional downstream immunohistological techniques, allowing quantification of nucleoside incorporation in all cardiac cell types.

**Figure:**
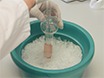


## Introduction

In recent years there has been an accumulation of evidence challenging the supposition that the heart is a terminally differentiated, post-mitotic organ^1,2^. However, quantification of cardiomyocyte turnover and regeneration remains challenging.

The difficulties in accurately identifying rare cardiomyocyte generation using standard immunohistochemical techniques are well reported^3^. In addition, the cellular source of cardiomyocyte generation remains uncertain with evidence for contributions by cardiomyocyte proliferation as well as by stem cell differentiation^4-6^. Therefore, the use of lineage tracing models which require knowledge of the cardiomyocyte progenitor phenotype is impossible and quantification of proliferation in a single population, including cardiomyocytes, is inappropriate. Furthermore a cardiomyocyte has the potential for endoreplication without karyokinesis (resulting in a polyploid cardiomyocyte) or karyokinesis in the absence of cytokinesis (resulting in a binucleated cardiomyocyte)^7,8^. The accurate quantification of cardiomyocyte turnover depends on the ability to distinguish between these events and true neo-cardiomyocyte generation. This creates unique complications because DNA replication and expression of cyclin-dependent kinases in cardiomyocytes do not exclusively demonstrate true cell division^9,10^.

To assist in the quantification of neo-cardiomyocyte generation, we have combined an established nuclei isolation technique, and immunological labeling of pericentriolar material 1 (PCM1) for cardiomyocyte nuclei identification as described by Bergmann *et al.*^7,11^ with novel methods of long term DNA labeling and ploidy analysis. PCM-1 is a centrosome protein that accumulates at the nuclear surface of differentiated, non-cycling myocytes. Previous studies have demonstrated that antibodies against PCM-1 specifically label cardiomyocyte nuclei^7,11^ and as such PCM1 has been used by a number of independent groups to identify cardiomyocytes^1,12,13^. In addition, we have demonstrated that PCM1 expression maps to genetically labeled cardiomyocyte nuclei in the TnT-cre transgenic mouse model^14^ (**Supplementary Figure 1**).

The protocol described here enables the accurate and sensitive identification of neo cardiomyocyte nuclei generation in the mouse heart, irrespective of the cellular origins (**Figure 1A **and** B**) while simultaneously excluding nucleoside labeling due to polyploidization from the analysis (**Figure 1C **and** D**). Although this method cannot control for cardiomyocyte binucleation, it allows a rapid and robust quantification of neo-cardiomyocyte nuclei which is required for the accurate quantification of cardiomyocyte turnover. Furthermore, it provides a rapid screening tool to assess potential changes in the dynamics of cardiomyocyte generation.

While DNA labeling usually involves 5-Bromo-2′-deoxyuridine (BrdU) as the thymidine analog, the protocol described here uses a 5-ethynyl-2'-deoxyuridine (EdU) based assay as it requires fewer processing steps for a more rapid through-put and does not require denaturing of the DNA for immuno-detection, making it compatible with other immunostaining protocols and thereby increasing the potential downstream applications of the method.


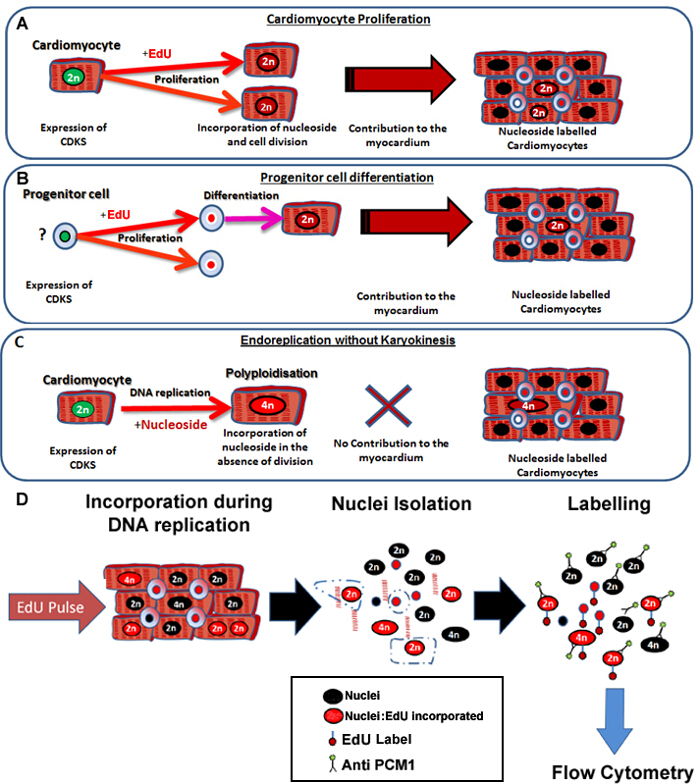
**Figure 1: Continuous pulsing with EdU labels neo-cardiomyocytes irrespective of their progenitors**. (**A**) EdU is incorporated into the DNA of cardiomyocytes during cell division. Proliferation in the cardiomyocyte population will result in an increase in, or replacement of cardiomyocytes and is therefore productive DNA synthesis (contributes to tissue maintenance and repair). (**B**) EdU is incorporated into the DNA of cardiac progenitor cells during cell division. This will be retained in the cell during differentiation to the cardiomyocyte lineage. This stem cell differentiation will also result in an increase in the number of cardiomyocytes and therefore contributes to tissue maintenance and repair. (**C**) Cardiomyocytes have the potential to undergo "non-productive" DNA replication resulting in increased cardiomyocyte ploidy, which is associated with cardiomyocyte hypertrophy and myocardial remodeling, but does not replace lost cardiomyocytes. The process of polyploidization differs from binucleation as it results in a cardiomyocyte with a single nuclei which contains four or more sets of two homologous chromosomes (>2N). (**D**) Following a continuous nuclei pulse, this protocol describes nuclei isolation and identification of the cardiomyocyte nuclei by PCM1 expression to allow quantification of both cardiomyocyte ploidy and EdU incorporation. PCM1 expression and EdU incorporation detected using flow cytometry. Please click here to view a larger version of this figure.

## Protocol

Animal work was authorized and approved by Newcastle University Ethics review board. All animal procedures were performed conforming to the guidelines from Directive 2010/63/EU of the European Parliament on the protection of animals used for scientific purposes.

### 1. EdU Administration

1. Dissolve EdU in sterile saline solution (0.9% w/v) at a final concentration of 12.5 mg/ml.
  1. Heat the solution to 40 °C and vortex in order to completely dissolve. Store enough EdU/Saline at 4 °C to inject all mice in the study allowing for 6 daily injections. Note: Typically over 6 mice are required for each experimental group. However, power calculations should be performed for independent studies.
2. Weigh individual mice. Calculate the required volume to administer 100 µg/g of EdU/saline solution to each mouse (8 µl of stock EdU solution per g).
3. Draw the appropriate volume of EdU/saline into insulin syringe with a 25 G needle.
4. Perform intraperitoneal (i.p.) injections while handling mice using approved Home Office techniques.
  1. Place a mouse on the cage lid. Gently pull back on the base of the tail and firmly grasp the mouse by the scruff.
  2. Expose the abdomen for injection by holding the mouse with the index finger and thumb near the base of the head and tipping the animal's head backwards toward the floor.
  3. Wipe the abdomen with 70% alcohol solution. Locate the animal's midline and lower right quadrant. Inject the EdU/Saline solution into the animal into the lower right quadrant.
  4. Inject mice with EdU/saline solution and record the time of day.
5. Repeat steps 1.2 to 1.4 at the same time of day, for 6 consecutive days. Note: As an EdU negative control is required to set the gating for flow cytometric analysis, inject an additional age, gender and experimentally matched mouse with an equivalent volume of saline without EdU. This will provide the required control (no EdU control). This control should be included for every study to allow for variations in batches of reagents and cytometer set up.

### 2. Harvesting Hearts

1. At 24 hr after the 6^th^ EdU injection, sacrifice mice using cervical dislocation (or an alternative Schedule 1 Home Office approved method).
2. Place sacrificed animal in a supine position and make skin incision from the mid abdomen to the diaphragm with surgical scissors.
3. Cut the diaphragm, and holding the sternum away from the body cavity, cut bilaterally to expose the heart.
4. Lift the heart slightly and cut the major blood vessels of the outflow tract to dissect the heart out of the thoracic cavity.
5. Immediately place each heart in to an individual 15 ml centrifuge tube containing 10 ml PBS chilled to 4 °C.
6. Transfer hearts to separate 10 cm Petri dishes, using a scalpel cut each heart in two in a sagittal orientation and wash the heart by gently squeezing with a pair of forceps. Then replace the PBS to remove as much blood as possible. Repeat this step twice.
7. Place both parts of each heart into the same individual 1.5 ml microcentrifuge tube.
8. Continue to step 3 or alternatively store specimens -80 °C as detailed in steps 2.9 and 2.10.
9. Using a 25 G needle, make a small hole in the microcentrifuge tube lid to prevent the lid from opening due to the expansion of gases when the heart is processed.
10. Label each microcentrifuge tube and place in a container of liquid nitrogen to freeze.

### 3. Nuclei Dissociation and Cardiomyocyte Nuclei Labeling

Note: This nuclei dissociation and cardiomyocyte nuclei labeling is adapted from that of Bergmann *et al.*^11^ Perform this procedure on all samples injected with EdU and saline injected (no EdU control) negative control. See **Table 1** for the recipe of solutions required to perform these steps.

1. For each heart to be analyzed, place 36 ml of 1% BSA/PBS solution into an ultracentrifuge tube, incubate for 30 min, discard the solution and then allow to air dry. Note: This tube is used in step 3.6.
2. Place individual frozen hearts onto a 10 mm dish on ice and mince using a scalpel allowing the hearts to defrost during the mincing process.
3. Place minced hearts into 15 ml of Cell Lysis Buffer in a 50 ml centrifuge tube and homogenize with a probe homogenizer for 15 sec at 25,000 rpm at room temperature.
4. Add a further 15 ml of Cell Lysis Buffer to the Centrifuge tube and transfer to a 40 ml dounce with a large clearance pestle. Perform 10 strokes with the pestle and filter through a 100 µm then 40 µm cell strainer into a 50 ml centrifuge tube.
5. Centrifuge for 10 min at 700 x g at 4 °C and remove the supernatant.
6. Resuspend the nuclear pellet in 25 ml of Sucrose Gradient Solution and layer the cell nuclei containing solution on top of 10 ml of fresh sucrose gradient solution in the ultracentrifuge tube prepared in 3.1.
7. Centrifuge at 13,000 x g for 1 hr at 4 °C using a swing out tube rotor.
8. Following the centrifugation, discard the supernatant and resuspend the nuclei pellet in 1,300 µl of nuclei storage buffer in a 1.5 ml microcentrifuge tube and label PCM1.
9. Remove 300 µl of the suspension to a new microcentrifuge tube and add to 700 µl of fresh nuclei storage buffer. Label this tube as ISO control.
10. Add anti-PCM1 at a final concentration of 8 µg/ml to the microcentrifuge tube labeled PCM1 and add Rabbit IgG isotype control antibody at a concentration of 8 µg/ml to the microcentrifuge tube labeled ISO control.
11. Incubate overnight at 4 °C. Following incubation, centrifuge at 700 x g for 10 min, discard the supernatant, replace with 1 ml of fresh nuclei storage buffer, resuspend and centrifuge again for 10 min at 700 x g. Discard the supernatant and replace with 1 ml of fresh nuclei storage buffer.
12. Add 1 µl of F(ab')2 Fragment of Goat anti-Rabbit IgG (H+L) antibody (FITC or equivalent green-fluorescent dye) to achieve a final concentration of 2 µg/ml. Note: The use of a F(ab')2 fragment antibody decreases the potential for non-specific labeling of immune cells including macrophages and B cells.
13. Wrap tubes in aluminum foil to protect from the light and incubate for 1 hr at 4 °C.

### 4. Detection of EdU Incorporation

1. Dilute 10x concentrated EdU reaction buffer 1:10 with deionized water.
2. Centrifuge the PCM-1 and ISO control labeled nuclei suspensions at 700 x g for 10 min, discard the supernatant and resuspend the nuclei pellet in 1 ml of 1% BSA/PBS solution. Wash the nuclei pellet twice.
3. After the final wash, discard the supernatant and replace with 100 µl of EdU fixative.
4. Wrap tubes in aluminum foil to protect from the light and incubate at room temperature for 15 min.
5. Wash the samples twice with 1 ml of 1% BSA/PBS solution, centrifuge at 700 x g and incubate with 1x saponin-based permeabilization solution at room temperature for 15 min.
6. Prepare 1x EdU labeling cocktail (**Table 2**). A minimum of 2 reactions will be required to include the no EdU control.
7. Add 500 µl of 1x EdU labeling cocktail directly to each sample already containing nuclei in 100 µl of 1x saponin-based permeabilization solution and incubate at room temperature protected from the light for 30 min.
8. Centrifuge at 700 x g and resuspend in 1 ml of 1% BSA/PBS and repeat this step two more times.
9. Centrifuge at 700 x g, discard supernatant and replace with 400 µl of DNA staining solution (see **Table of Materials**).

**Table:** 

**Name of the reagent**
**1. Cell Lysis Buffer**
0.32 M sucrose
10 mM Tris-HCl (pH = 8)
5 mM CaCl_2_
5 mM magnesium acetate
2.0 mM EDTA
0.5 mM EGTA
1 mM DTT
**2.Sucrose Gradient Solution**
2.1 M sucrose
10 mM Tris-HCl (pH = 8)
5 mM magnesium acetate
1 mM DTT
**3. Nuclei storage buffer (NSB)**
0.44 M sucrose
10 mM Tris-HCl (pH = 7.2)
70 mM KCl
10 mM MgCl_2_
1.5 mM spermine

**Table 1: Nuclei isolation buffer ingredients.** Recipe for all Buffers used in protocol section 3 (Nuclei dissociation and cardiomyocyte nuclei labeling).

### 5. Flow Cytometric Analysis

Note: Perform Flow cytometry on isolated nuclei as described previously^7,11,15^.

1. First, create a plot describing Forward Scatter (FSC) and Side Scatter (SSC) to allow the discrimination of nuclei from debris (**Figure 2A**).
2. Create a plot to discriminate single nuclei from aggregates (**Figure 2B**) by plotting 4',6-diamidino-2-phenylindole) (DAPI) area (3-405/450/50-A) versus height (33-405/450/50-H). Ensure that the 3-405/450/50-A signal is collected on the linear scale to allow the correct determination of DNA content.
3. Create a plot describing 488/535/30-A (PCM-1) vs SSC and display only events from the singlet gate created in 5.2 in order to assess PCM-1 expression in the singlet population.
4. Run the Isotype control sample in order to establish a PCM-1 positive gate (**Figure 2C**). Run a small amount of PCM1 labeled sample to verify the PCM1 expressing population and gating position.
5. Create a plot describing SSC-A vs 405/450/50-A (to detect DAPI). In this plot, display only singlet PCM1 expressing nuclei, using the gates created in 5.3. Use this plot to create an additional hierarchal gate that contains only 2N nuclei (**Figure 2D**).
6. Create a plot that describes 488/535/30-A vs 1-633/660/20-A to allow the identification of EdU labeling of the PCM1 expressing cardiomyocyte population. Display only nuclei which are singlet, PCM1+ and 2N using the above gating.
7. Run heart sample from no EdU control to set the EdU positive gate (**Figure 2E**). Create the appropriate gate for EdU+ cells.
8. Record and quantify singlet, PCM-1 expressing, 2N nuclei that have incorporated EdU.
9. Calculate this population as a percentage of total singlet, PCM1+ nuclei. This will provide the rate of neo-cardiomyocyte nuclei generation, during the 7 day EdU pulse period, as a percentage of total cardiomyocyte nuclei. Note: As DAPI binding to DNA is stoichiometric the fluorescence intensity is proportional to the amount of DNA. Determine ploidy based on the intensity of the 405/450/50-A signal, as described previously^7^.
10. To evaluate the ploidy within the total cardiomyocyte population use the plot established in 5.5 and a gating strategy based on DNA concentration^7^.

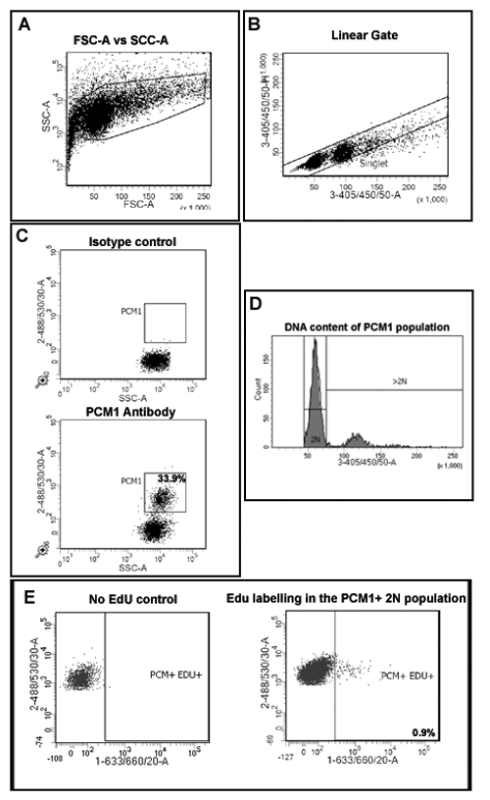
**Figure 2: Gating strategy to quantify cardiomyocyte EdU incorporation and ploidy.** (**A**) Nuclei are discriminated from debris based on forward scatter (FSC) and side scatter (SSC). (**B**) A linear gate is created and the singlet nuclei population is identified based on DAPI labeling and the 405/450/50 height vs area signal. (**C**) Fluorescent gating allows the separation of cardiomyocyte nuclei (PCM-1-positive) and non-cardiomyocyte (PCM-1-negative) nuclei from heart tissue. (**D**) Intensity of DAPI signal is used to determine DNA concentration and thereby nuclei ploidy of the PCM1+ cardiomyocyte population. In mouse >80% of cardiomyocyte nuclei are diploid (2n). (**E**) Fluorescent gating allows the separation of 2N, cardiomyocyte nuclei which have incorporated EdU (PCM+/EDU+ = 0.9%) from 2N, cardiomyocytes which have not incorporated EdU (PCM+EDU-). All steps are detailed in the protocol 5.0. Example shown from mdx^-/y ^mice on the C57/BL10 background. Please click here to view a larger version of this figure.

## Representative Results

This method allows the quantification of increased EdU incorporation due to cardiomyocyte nuclei division while controlling for increased polyploidization. Using a BrdU based assay, we previously demonstrated that the mammalian heart responds to chronic cardiomyocyte loss, in the *mdx* mouse model of Duchenne muscular dystrophy, with the regeneration of new cardiomyocytes. We have used the methods described here to further validate our published findings and to demonstrate the utility of the protocol. As per the protocol described; adult (12 week old) *mdx* and control bl10 mice were pulsed with one daily injection of EdU for 7 days, reported previously to allow statically analysis between experimental groups^5,13^. Immunohistological analysis of pulsed hearts demonstrates the presence of PCM1 expressing cardiomyocytes which have incorporated EdU (**Figure 3A**). However, the data obtained using immunohistological methods can be misinterpreted, as other cell types which have incorporated EdU can be misidentified as cardiomyocytes. An example of this is demonstrated in **Supplementary Figure 1A **and** B**. Furthermore immunohistological methods do not distinguish polyploidization from nuclear division. The flow cytometry analysis detailed in this protocol enables the rapid quantification of cardiomyocyte nuclear division in *mdx* and control mice, while excluding cells which had incorporated EdU due to polyploidization. Similar to previously published data^13^, analysis revealed an increase in the rates of neo-cardiomyocyte nuclei generation in the *mdx* mouse hearts compared to age matched controls (1.2% versus 0.17%; **Figure 3B **and** C**).


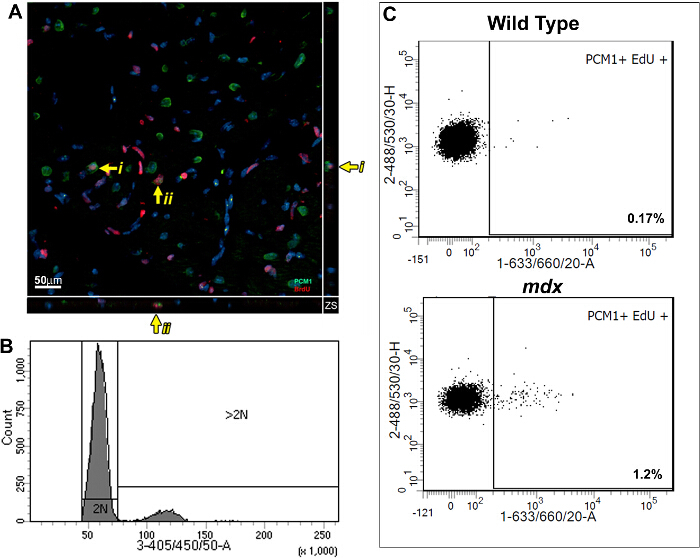
**Figure 3: Quantification of EdU labeled cardiomyocytes in wildtype and *****mdx***** hearts. **(**A**) Image of Z-stack projections taken from 40 µm thick sections of *mdx* hearts. PCM-1 expressing cardiomyocyte nuclei (green) which has incorporated EdU (red). Yellow arrow indicates EdU labeled cardiomyocytes. i and ii indicates individual EdU labeled cardiomyocytes shown in the Z-plane. Nuclei labeled with DAPI (blue) (**B** and **C**) Flow cytometry of isolated nuclei showing representative plots from 12-13 week old wild type (C57/BL10) and *mdx* mice (mdx^-/y ^on the C57/BL10 background). (**B**) Histogram showing DAPI intensity and discrimination between 2N and >2N nuclei. (**C**) Plots showing EdU incorporation in the 2N cardiomyocyte nuclei population when analyzed as detailed in this protocol. For all mice Edu was administered for 1 week from 12 weeks of age. Please click here to view a larger version of this figure.

**Supplementary Figure 1: PCM-1 is a specific marker for cardiomyocyte nuclei. **Using the protocol described nuclei were isolated from the double transgenic mouse produced by crossing TNT-cre mice^14^ with the ROSA^nT/nG^cre sensitive reporter line^16^. In the resulting mouse *cre* activation under the control of Troponin T (TNT) promoter leads to irreversible expression of GFP with is localized to the nuclei. Nuclei labeled with Isotype control (Iso) demonstrates the ability to identify the GFP expressing cardiomyocyte nuclei population (488/530/30-A). Labeling of nuclei with anti-PCM1 (secondary antibody detected by 633-660/20-A) demonstrates the correlation of PCM1 expression with the GFP expressing cardiomyocyte nuclei population. In this example 98.8% of cardiomyocyte nuclei as identified by TNT promoter activity, are labeled by PCM1 antibody. Please click here to download Supplementary Figure 1.

**Supplementary Figure 2: Challenges for quantifying cardiomyocyte renewal by immunohistological techniques.** (**A**) 2D Image shows two PCM-1 expressing cardiomyocyte nuclei (green) which appear to be also labeled for EdU (red). Yellow arrows indicate what appears to be cardiomyocytes which have incorporated EdU. This is due to the apparent co-localization of PCM1 expression and EdU labeling when visualized using 2-dimentional imaging. (**B**) A 3-dimensional projection of this image identifies the EdU labeled cells are not cardiomyocytes but are non-cardiomyocyte cells overlying PCM1 expressing cardiomyocyte nuclei. C57/BL10 mice at 12 weeks of age used for experiments. Please click here to download Supplementary Figure 2A. Please click here to download Supplementary Figure 2B.

**Table:** 

**Reaction Components**	**Number of reactions**
2	5
PBS	875 µl	2.19 ml
Copper protectant	20 µl	50 µl
Fluorescent dye picolyl azide	5 µl	12.5 µl
Diluted reaction buffer prepared in 4.1	100 µl	250 µl
Total reaction volume	1 ml	2.5 ml

**Table 2: EdU cocktail ingredients**. Recipe for EdU labeling cocktail required in protocol section 4 (Detection of EdU incorporation).

## Discussion

To accurately quantify cardiomyocyte turnover and regeneration assays must distinguish between true cardiomyocyte generation and nonproductive DNA division. Many studies continue to simply ignore these nonproductive events, quantifying cardiomyocyte proliferation solely via the expression of cyclin kinesis and cell cycle markers. To date a single method that allows the accurate quantification of cardiomyocyte turnover while controlling for these nonproductive events remains allusive. In particular it remains difficult to account for cardiomyocyte polyploidization which contributes up to ~65% of cardiomyocyte DNA replication^13^. Therefore to assist in the accurate quantification of cardiomyocyte generation we have developed a protocol that allows the robust quantification of the rates of neo cardiomyocyte nuclei while excluding DNA replication that results in increased ploidy. Although this protocol cannot discrimination between neo-cardiomyocyte generation and cardiomyocyte bi-nucleation, it can be used rapidly and accurately calculate the upper limit (accounting for ploidy) of cardiomyocyte generation. This protocol therefore provides a screening tool to assess potential changes in the rates of cardiomyocyte generation and polyploidization in disease models or to evaluate the potential efficiency of therapeutics. Once changes in the rate of neo-cardiomyocyte nuclei generation are identified using this protocol subsequent studies can be used to ascertain if this due to changes in cardiomyocyte generation of cardiomyocyte nucleation number, as described previously^2,13,17,18^. These include the use of histological quantification cardiomyocyte nucleation dynamics during the pulse period or analyses of tissue sections obtained from EdU pulsed animals in to compare EdU incorporation in the mononucleated and multinucleated cardiomyocyte populations.

Due to the low levels of cardiomyocyte turnover this protocol uses multiple injections of EdU over a 7 day period. This also allows the "chasing" of all potential cellular sources of cardiomyocyte generation and permits quantification of cumulative cardiomyocyte nuclei generation over this time period. Depending on the study, this timeframe may be adjusted to suit the predicted levels of cardiomyocyte generation. For the accurate quantification of EdU incorporation in cardiomyocyte nuclei, it is imperative that there is no non-specific labeling of the nuclei with the secondary antibody used to detect PCM-1 reactivity. It would therefore be prudent to undertake additional secondary antibody titration experiments in order to optimize this aspect of the protocol, particularly if a secondary antibody other than that suggested in this protocol is to be used. The protocol described here uses PCM-1 expression to identify cardiomyocyte nuclei. While this is an established cardiomyocyte marker, alternative markers can be used to validate data; these include antibodies specific for cardiac Troponin T which has been identified as partly localized in cardiomyocyte nuclei^1^. Similarly, alternative nuclear localized proteins may be used to identify and quantify EdU incorporation in nuclei populations other than that of the cardiomyocytes. It is important that all cardiomyocyte nuclei that are actively undergoing mitosis are excluded from the analysis, as the fate of this DNA synthesis is unknown and may result in either cell division or increased ploidy. PCM1 is disassembled during the M phase of the cell cycle therefore cardiomyocytes undergoing mitosis will not be identified by PCM1 expression. In addition, all nuclei in the s phase of the cell cycle should be excluded from subsequent analysis. This can be achieved by gating out all nuclei with a DAPI intensity above the 2N population including those with a DAPI intensity between the 2N and 4N populations.

Although it is increasingly accepted that the heart has the capacity to replace cardiomyocytes during normal aging and following acute injury, the source and degree of this potential remains controversial. In addition, disparate rates of cardiomyocyte turnover have been reported^1,7,20-22^. This may be in part due to the difficulties in accurately identifying and quantifying neo-cardiomyocyte generation^19^. To date the majority of studies have relied only on the use of histological analysis and the identification of cardiomyocytes via the expression of cytoplasmic proteins, including proteins of the sarcomere, for the quantification of cardiomyocyte turnover and renewal^2,4,23,24^. The use of these methods to detect the expression of proliferation markers, or as demonstrated here, the incorporation of thymidine analogues can easily result in the misidentification of other cardiac cell types as cardiomyocytes. While the use of 3D confocal imaging can help to alleviate these problems these methods are expensive and time consuming. Interestingly, the protocol described here demonstrates neo-cardiomyocyte nuclei generation occurs at a rate of 0.17% per week. This is consistent with other flow cytometry based studies demonstrating weekly turnover rates of up to 0.13%^5^. Although it is tempting to extrapolate annual turnover rates based on this data, as in previous studies^2,5,25,26^, this is inappropriate as the rates of turnover are dynamic during life time of an animal^13^.

This method has a number of potential applications including assessing the potential therapeutics to enhance cardiomyocyte regeneration or investigating the effects of cardiac disease on cardiomyocyte turnover and the rates of cardiomyocyte polyploidization.

## Disclosures

The authors have nothing to disclose.
